# Paratuberculosis, Animal Welfare and Biosecurity: A Survey in 33 Northern Italy Dairy Goat Farms

**DOI:** 10.3390/ani13142346

**Published:** 2023-07-18

**Authors:** Alessandra Gaffuri, Filippo Barsi, Edoardo Magni, Stefania Bergagna, Debora Dellamaria, Matteo Ricchi, Livia De Paolis, Giorgio Galletti, Norma Arrigoni, Valentina Lorenzi, Francesca Fusi, Alice Prosperi, Chiara Garbarino

**Affiliations:** 1 Istituto Zooprofilattico Sperimentale della Lombardia e dell’Emilia Romagna “Bruno Ubertini”, 25124 Brescia, Italy; alessandra.gaffuri@izsler.it (A.G.); matteo.ricchi@izsler.it (M.R.); giorgio.galletti@izsler.it (G.G.); norma.arrigoni@izsler.it (N.A.); valentina.lorenzi@izsler.it (V.L.); francesca.fusi@izsler.it (F.F.); alice.prosperi@izsler.it (A.P.); chiaraanna.garbarino@izsler.it (C.G.); 2 Italian National Reference Centre for Paratuberculosis, Istituto Zooprofilattico Sperimentale della Lombardia e dell’Emilia Romagna “Bruno Ubertini”, 29027 Piacenza, Italy; 3 Official Veterinary Service, ATS Montagna, 23100 Sondrio, Italy; edoardo.magni101@gmail.com; 4 Istituto Zooprofilattico Sperimentale del Piemonte Liguria e Valle d’Aosta, 10154 Torino, Italy; stefania.bergagna@izsto.it (S.B.); livia.depaolis@izsto.it (L.D.P.); 5 Istituto Zooprofilattico Sperimentale delle Venezie, 35020 Padova, Italy; ddellamaria@izsvenezie.it; 6 Italian National Reference Centre for Animal Welfare, Istituto Zooprofilattico Sperimentale della Lombardia e dell’Emilia Romagna “Bruno Ubertini”, 25124 Brescia, Italy

**Keywords:** *Mycobacterium avium* subsp. *paratuberculosis*, MAP, dairy goats, serology, biosecurity, animal welfare assessment

## Abstract

**Simple Summary:**

Paratuberculosis is a chronic incurable bacterial infection widespread all over the world in ruminants. The disease impacts animal health and welfare and causes significant economic losses in animal productions. This survey investigated the spread of paratuberculosis in northern Italian dairy goat farming by serological testing. Contextually, a welfare and biosecurity assessment through a standardized protocol was conducted. More than half (19 out of 33, 58%) of the investigated farms were infected, with a mean intra-herd prevalence of 7.4%. Welfare assessment showed quite favorable average results, although in 24% of the farms the welfare level was poor. On the contrary, 58% of the farms showed an unsatisfactory biosecurity level. Our results provide information on the spread of paratuberculosis in dairy goat farms of northern Italy. For this relevant disease, official prevalence data in goat breeding are still scarce. Moreover, the present work highlighted the low level of biosecurity measures implemented by the farmers.

**Abstract:**

Paratuberculosis is a notable infectious disease of ruminants. Goats appear to be particularly susceptible. The survey aimed to investigate the spread of paratuberculosis in Italian goat farming and evaluate whether the presence of the disease could be influenced by welfare and biosecurity deficiencies. A serological survey for paratuberculosis in 33 dairy farms in northern Italy was conducted. Contextually, animal welfare and biosecurity were assessed, using a standardized protocol of 36 welfare indicators and 15 biosecurity indicators which assigns to each farm a welfare and biosecurity score from 0 (any application) to 100% (full application). An overall result of less than 60% was considered insufficient. Nineteen farms (58%) tested positive for paratuberculosis, with a mean intra-herd seroprevalence of 7.4%. Total welfare ranged from 39.56 to 90.7% (mean 68.64%). Biosecurity scores ranged from 10.04 to 90.01% (mean 57.57%). Eight farms (24%) showed poor welfare conditions (welfare score < 60%) and 19 (58%) an unsatisfactory biosecurity condition (biosecurity score < 60%). With respect to the explorative character of the study, an indicative association between seven welfare and biosecurity indicators and paratuberculosis seropositivity was identified. The presence of paratuberculosis in northern Italy dairy goat farms was confirmed. The welfare and biosecurity assessment protocol proved to be an accurate tool, capable of identifying critical points for managing health, welfare and productivity.

## 1. Introduction

Paratuberculosis is a chronic incurable enteritis of ruminants caused by *Mycobacterium avium* subsp. *paratuberculosis* (MAP) [1]. The transmission primarily occurs through the ingestion of infected feces. 

The disease is important because of its impact on the economy, on the animal welfare and for public health in general [2]. Among domestic ruminants, goats appear to be particularly susceptible [3]. It has been observed that goats are naturally more susceptible to MAP infection than sheep and cattle and may play a more important role than sheep in the transmission and maintenance of the disease [2,4,5]. In goats, the onset of clinical signs is most common between two and three years of age, whereas subclinical infection is most often seen in the early years [2,6]. Indeed, paratuberculosis in this species is insidious and symptoms are usually not clearly evident. As a consequence, it is often diagnosed only at the latest state of disease when it has spread to most animals of the flock. Infected individuals often do not show diarrhea but non-specific signs as weight loss, exercise intolerance and decreased milk production [7]. Sardaro et al. [8] reported that economic losses and consequent profit inefficiency caused by the disease in breeding of small ruminants are due to decreased milk production, diagnostic and disease control costs, culling of affected animals and low carcasses values at slaughter. Surveillance and control of paratuberculosis can be of critical importance in some developing countries where small ruminants play a vital role in the livelihood of poor communities, as well in worldwide disadvantaged areas and in the increasing sector of intensive goat breeding [9,10]. 

Another important reason to investigate the disease in animals is related to the detection of MAP in humans affected by different chronic diseases, such as Chron’s disease. These observations suggested a hypothetical zoonotic role for MAP that thus far, has not been confirmed or denied [11]. In this context, since several studies detected MAP in goat cheeses, often made from raw milk [12,13,14,15], from a health-risk point of view, contamination with MAP of foods of animal origin should be prevented. 

According to the Regulation (EU) 2018/1882 [16], paratuberculosis is subjected to surveillance in cattle, buffaloes, sheep, goats, camelids and cervids. 

Although the Regulation (EU) 2018/1882 clearly reports the obligation to notify the disease, because of scarce knowledge or difficulties in diagnosing subclinical infections [5], the disease is often underreported [2].

All over the world, paratuberculosis has been reported in goats [2]. In Europe, Nielsen and Toft [17] reported an inter-herd prevalence of infected goat herds over 20%. 5. Jiménez-Martín et al. [5] performed a cross-sectional investigation on 83 sheep farms and 70 goat farms in Andalusia (southern Spain) and detected an apparent seroprevalence of 90% in goat flocks and 66.3% in sheep flocks. In the same study, the estimated individual true seroprevalences were 8.4% for sheep and 25.2% for goats.

In Italy, goat farming is still considered marginal despite the fact that the presence of the species is recorded throughout the country (about 1,000,000 heads were reared in 2022, of which 300,000 dairy goats (https://www.vetinfo.it/, accessed on 11 April 2023) and it is expanding.

In Italy, paratuberculosis in goats was reported in Tuscany region (central Italy) [18] and Apulia region (southern Italy) [10]. This last epidemiological study was carried out in 419 semi-extensive dairy goat, sheep and mixed flocks and reported a true seroprevalence at flock level ranging from 63.8 to 92.4% in flocks with different species of small ruminants. Moreover, the same study reported, at individual level, statistically significant higher seroprevalence in goats, confirming the great sensitivity of this species to MAP infection [10]. To the authors’ knowledge, no other published studies are available, underlining the scarcity of these data for most of the Italian regions, especially those where the breeding of goats represents an important local industry because of the cheese production and other typical products. 

Notably, for its impact on goat health, paratuberculosis is one of the diseases–together with caseous lymphadenitis and caprine arthritis encephalitis–specifically considered for their overall effect on goat welfare [19]. On the other hand, management deficiencies in terms of animal welfare and biosecurity could lead to the introduction and spread of the disease on the farm. In fact, the application of biosecurity measures is directed to prevent the introduction and spreading of MAP infection in the farm, whereas welfare assessment focuses on farm management measures and structural characteristics, both potentially impacting on the spread and progression of the disease. 

In the last decade, the assessment of animal welfare at farm level received increasing attention but, for some species, such as goat, there is still no specific legislation both at European and Italian level. Since 2015, the Italian Reference Centre for Animal Welfare, located at Istituto Zooprofilattico Sperimentale della Lombardia e dell’Emilia Romagna (IZSLER-CReNBA), has implemented a specific protocol for the on-farm animal welfare and biosecurity assessment in dairy goats. 

The aim of this study was to carry out an exploratory investigation on the occurrence of paratuberculosis in Italian goat farming and to evaluate whether the presence of paratuberculosis could be influenced by herd management in terms of welfare and biosecurity measures. For this purpose, we conducted a survey in 33 dairy goat farms spread across four regions in northern Italy providing welfare and biosecurity assessment data by a specific protocol and collecting data about paratuberculosis status by ELISA test.

## 2. Materials and Methods

This study was carried out in the frame of a research project funded by the Italian Ministry of Health from 2019 to 2021. Thirty-three dairy goat farms were involved in this study. Farmers were contacted through veterinary practitioners who already had relationships with the laboratories. Farmers showing interest in the project voluntarily joined it and their farms were included in the sampling. Enrolled flocks were located in four different regions of northern Italy: Piedmont (5), Trentino-Alto Adige (3), Emilia-Romagna (5), and Lombardy (20). 

Selected farms were visited once during 2019. In the same occasion, blood samples were taken, and animal welfare and biosecurity measures inspections were performed by trained veterinarians using the specific protocol of IZSLER-CReNBA (see Section 2.2). 

The milk yield of each farm was recorded for one year starting from the first visit, so the average milk production per head per day was calculated.

### 2.1. Serological Assay

Samples for serological analysis were collected by the Official Veterinary Services in the frame of mandatory regional brucellosis control plans. Therefore, it was not necessary to collect additional samples for the present investigation. Blood samples were taken by jugular venipuncture into vacutainer tubes without anticoagulant. Samples of the animals over 12–18 months of age were stored at refrigeration temperature (4 °C) and analyzed in a few days. 

Serological analysis was performed by an ELISA commercial kit (ID Screen^®^ Paratuberculosis Indirect, ID-vet, Montpellier, France), according to the manufacturer’s instructions: inconclusive (0.6 < S/P ratio < 0.7) and positive (S/P ≥ 0.7) samples from the screening test were submitted to confirmatory testing by an additional ELISA commercial kit (ID Confirmation^®^ Paratuberculosis Indirect, ID-vet, Montpellier, France). Samples with a S/P ratio of 0.7 or above in the confirmation test were considered positive. In goats, the sensitivity of ELISA ranged from 63 to 100%, while the specificity of ELISA generally ranged from 92 to 100% [20].

### 2.2. Welfare and Biosecurity Assessment

On-farm animal welfare and biosecurity assessments were performed using the specific IZSLER-CReNBA protocol for dairy goat farms. This protocol was developed in 2015 in the frame of another research project called “RuminantWelfare”, following the method described in Bertocchi et al. [21] and Lorenzi et al. [22]. The protocol included animal-based measures (ABMs) and resource-based indicators (also referred to as non-animal-based measures-N-ABMs) [21]. Briefly, these welfare indicators were selected based on the available scientific literature [23,24], on the European legislation (Council Directive 98/58/EC) [25] and its Italian transposition (Legislative Decree 26 March 2001, n.146), on the AWIN welfare assessment protocol for goats [26] and on an expert knowledge elicitation (EKE) [27]. In particular, the opinion of 14 Italian veterinarians was gathered during an EKE in order to characterize a set of management and housing factors potentially associated with negative or positive welfare outcomes in dairy goats kept in loose housing systems in Italy [27]. Experts were asked to weigh the potential negative or positive impacts of each factor in relation to five welfare categories (udder health, metabolic needs, locomotion and foot health, integument integrity and behavior) by estimating the magnitude (scoring scale from 0–none–to 3–high) and the likelihood (from 0% to 100%) of the negative or positive welfare consequences that could be associated with the exposure of the animals to each of proposed factors and to rate the certainty in relation to the likelihood value they provided (scoring scale: high, medium, low). The data obtained from the EKE were used to set the final protocol and to weight the different indicators. 

The welfare protocol includes 36 parameters divided into three areas: “Area A: farm management and staff training” (indicators from 1 to 11), “Area B: housing” (indicators from 12 to 24), and “Area C: animal-based measures” (indicators from 25 to 36) (Table 1).

Additionally, the protocol includes 15 indicators on relevant aspects of biosecurity in ruminant breeding (Table 2) [28,29]. 

Currently, this protocol is part of the ClassyFarm system of the Italian Ministry of Health (https://www.classyfarm.it/check-list/, accessed on 11 April 2023) and it is voluntarily applied in intensive, semi-intensive and semi-extensive dairy goat farms at national level.

By means of the described protocol, data were collected by six trained veterinarians who have previously attended a specific training course in IZSLER and routinely applied the assessment method in their field activity, with the aim to ensure a high intra- and inter- observer reliability. For each indicator, the evaluator assigned a score based on a 2 or 3-point scale scoring system, where 1 indicated an insufficient status or high level of risk, 2 and 3 indicated, respectively, an acceptable and excellent status of the indicator, or low level of risk. An insufficient assessment corresponds to clear negative evidence or measures below the target levels set by the system, an acceptable assessment corresponds to clear adequate evidence or measures that meet the target levels, whereas an excellent assessment corresponds to clear positive evidence or measures above the target levels. A resulting value for each section was calculated as described in [30,31]. Briefly, each indicator threshold (i.e., 1, 2 or 3) had a different ‘weight’ according to its potential impact on animal welfare and health, these weights were assigned by means of the previously described EKE. A value for each of the three welfare areas and for the biosecurity section was calculated per farm by summing up the score of each indicator, according to the answer assigned on farm [31]. The final welfare score was calculated as reported in [30], considering a 50% contribution by Areas A and B and 50% by Area C. All these values were expressed as a percentage from 0 to 100, where 0% indicated lack of any welfare/biosecurity measure and 100% indicated their full application. An overall result of less than 60% was considered insufficient. 

After obtaining the overall rating for welfare and biosecurity, a statistical evaluation was carried out in order to evaluate if the scores assigned to each indicator had a correlation with the presence of paratuberculosis at farm level.

### 2.3. Data Analysis

Descriptive analysis was performed for lactating goats, milk production, welfare and biosecurity assessment, and for each indicator. Continuous variables are shown as mean ± standard deviation (SD), and selected percentiles. Categorical variables (scoring for the indicators) are presented as absolute and relative frequency. Statistical analysis using the Chi-Square test was also provided for categorical variables, in order to explore potential association with paratuberculosis status. All the analyses were performed using R 4.0.2 [32]. 

## 3. Results

The data relative to number of lactating goats of the flocks, recorded at the moment of the sampling and welfare assessment, as well as their mean year-long milk production (expressed as kilograms per head per day) are summarised in Table 3. 

An intensive type of management was adopted by 25 farms (76%), whereas eight farms (24%) adopted a semi-intensive management. The represented breeds were Chamois Coloured goat, Saanen, Roccaverano goat, Murciana, Nubian, Nera Verzasca and crossbreed (Table S1). 

### 3.1. Serological Assay

**Figure 1:**
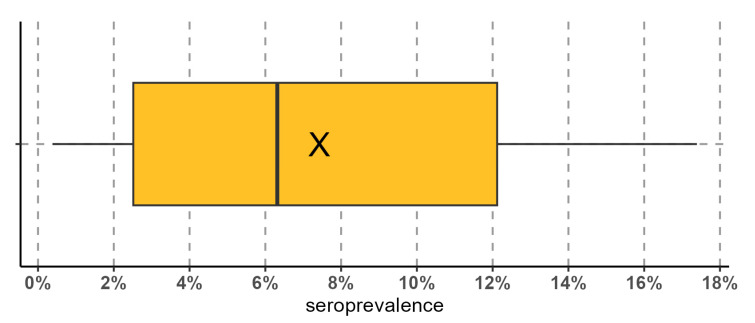
Boxplot of the intra-herd paratuberculosis seroprevalence. The mean value is represented by the “X”.

A total of 164 out of 4431 analysed samples tested positive for paratuberculosis. A total of 19 out of 33 (58%) farms were infected (with at least one seropositive animal), with a mean intra-herd apparent seroprevalence of 7.4% (range: 0.4–17.4%; Figure 1). When distinguishing on the basis of the type of management adopted, 14 out of 25 (56%) intensive farms and 5 out of 8 (62.5%) semi-intensive farms were infected. 

### 3.2. Welfare and Biosecurity Assessment

The results obtained from welfare and biosecurity assessment in the sampled farms are summarized in Table 4. Overall, total welfare ranged from 39.56 to 90.7% (mean = 68.64%, SD = 12.61%); in detail, data ranged from 41.29 to 96.16% (mean = 67.92 %, SD = 15.25%) for Area A, from 37.21 to 93.59% (mean = 60.36%; SD = 13.65%) for Area B, and from 30 to 100% (mean = 70.41%; SD = 15.57%) for Area C. Biosecurity data ranged from 10.04 to 90.01% (mean = 57.57%, SD = 19.25%). 

**Figure 2:**
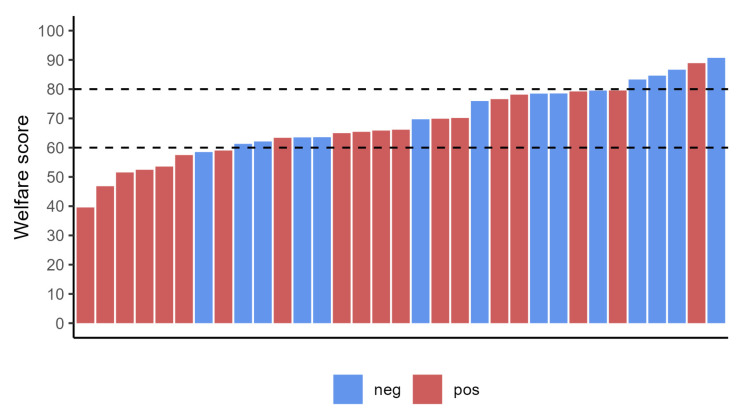
Welfare scores in paratuberculosis positive and negative flocks. The dotted lines indicate the thresholds among insufficient (below 60%), medium (61–80%), and high score (over 80%).

**Figure 3:**
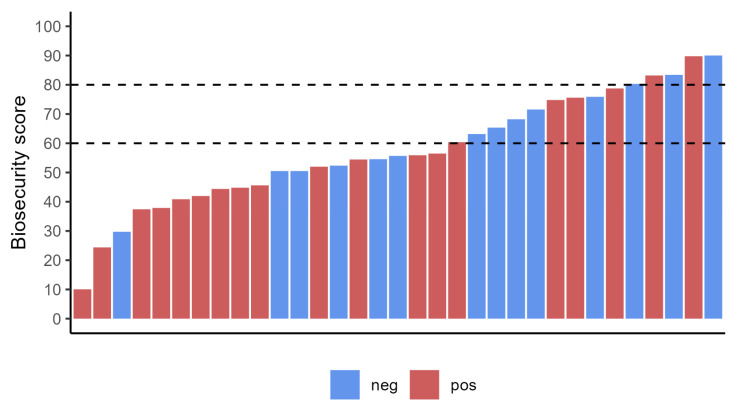
Biosecurity scores in paratuberculosis positive and negative flocks. The dotted lines indicate the thresholds among insufficient (below 60%), medium (61–80%), and high score (over 80%).

From the compilation of the welfare checklist, regarding “total welfare”, 24% of the farms (8 out of 33) obtained an insufficient score (below 60%), 61% (20/33) a medium score (between 60 and 80%), and 15% (5/33) a high score (over 80%; Figure 2). From the compilation of the biosecurity checklist, 58% (19/33) of the enrolled flock showed insufficient measures of biosecurity, 27% (9/33) obtained a medium score, and 15% (5/33) a high score (Figure 3).

**Figure 4:**
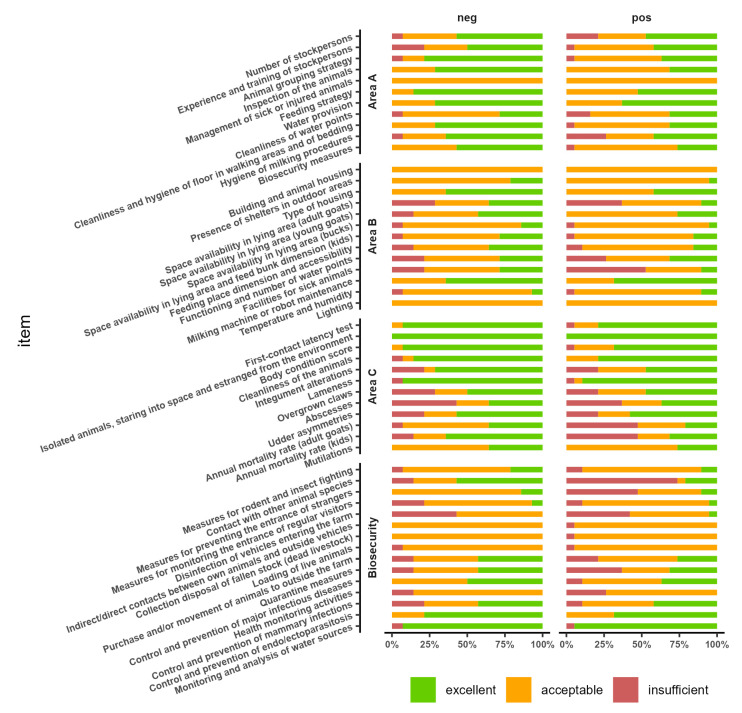
Percentage distribution of the scores assigned to each indicator in paratuberculosis positive and negative flocks.

In Figure 4 we reported the percentage distribution of the scores assigned for each indicator, according to the presence or absence of the disease in the farms.

Table 5 shows the result of statistical analysis (Chi-square test). In general, there is no strong evidence of an association between the indicators and paratuberculosis status. The analysis suggests a potential association with paratuberculosis status (*p*-value < 0.10) for the indicators “Animal grouping strategy”, “Inspection of the animals”, “Cleanliness and hygiene of floor in walking areas and of bedding”, “Space availability in lying area (young goats)”, and “Annual mortality rate (adult goats)” from the animal welfare assessment, “Contact with other animal species” and “Measures for preventing the entrance of strangers” from the biosecurity assessment. 

## 4. Discussion

In the present study, we carried out a serological survey for paratuberculosis on 33 dairy goat farms, located in four regions of northern Italy. Contextually, animal welfare and biosecurity assessment were carried out on the same farms, using a protocol developed by IZSLER-CReNBA and based on the use of animal-based measures (ABMs) and resource-based indicators. 

More than half (58%) of the investigated farms were seropositive for paratuberculosis, with mean intra-herd seroprevalence of 7.4%. In all the seropositive farms, at least one seropositive animal was confirmed to be infected by testing a fecal sample by a qPCR targeting the IS900 sequence of MAP [33]. Considering the limitations of the serological test, with a diagnostic sensitivity varying according to the stage of the disease [20], the prevalence of seropositive animals has been probably underestimated. The data shown confirmed, in agreement with those worldwide reported, the high diffusion of the disease in the northern Italy dairy goat farms. In 2023 the Italian Ministry of Health, in the frame of application of the Regulation (EU) 2016/429 [28] (“Animal health law”), included goat in the “National guidelines for the control of paratuberculosis” [34]. 

Regarding the welfare assessment, favorable average scores were found both in the overall score and for the three areas individually taken (Table 4). Out of the 33 farms considered in the present study, 25 (76%) obtained a positive score from the overall welfare assessment while eight farms did not reach the threshold score of 60%. In infected flocks, seven out of 19 (37%) showed an insufficient total welfare score (Figure 2). Considering the mean score of the three areas, the “housing” (Area B) had a mean insufficient score (56.91%), while “farm management and staff training” (Area A) and ABMs (Area C) showed, respectively a mean score of 61.23 and 67.11%, above the acceptability threshold. In non-infected flocks, registered mean scores for the three areas were higher: 76.99%, 65.03 and 74.89% for Area A, B and C, respectively. 

In order to explore potential association between paratuberculosis status and the 36 welfare indicators provided by the protocol, we compared the scores assigned to paratuberculosis-positive farms with the negative ones. According to the statistical analysis (Table 5), five indicators showed an association with paratuberculosis positivity: (i) “Animal grouping strategy”, (ii) “Inspection of the animals”, (iii) “Cleanliness and hygiene of floor in walking areas and of bedding”, (iv) “Space availability in lying area (young goats)”and (v) “Annual mortality rate (adult goats)” from the animal welfare assessment.

Regarding animal grouping, this indicator evaluates the separation into homogeneous groups from six months of age. Although this represents a good practice for the management of infectious diseases [35], the association highlighted may be misleading as the indicator does not take into account the early separation of the kids from the mother, a cornerstone of paratuberculosis management in the farm [7]. A regular inspection of the animals by the stockpersons is important for the detection of symptomatic animals and therefore allows the improvement of biosecurity and management practices. Cleanliness of facilities is one of the most important factors related to paratuberculosis, due to its fecal–oral transmission [35,36]. Regarding the space availability in lying area, the higher density of animals may increase horizontal transmission, mainly due to closer contact between goats. The statistical analysis underlined an association for young goats and not for adults, even if this category is the major source of the infection. This is especially true during the lambing season, since goats usually give birth all together in one pen and kids can easily come into contact with feces of adults before being separated from them. With respect to the annual mortality rate, even if not all the deaths can be referred to paratuberculosis, it is important evidence of the health problems and mismanagement inside the farms [5]. 

Concerning the biosecurity assessment, more critical issues were found compared to welfare. Nineteen out of 33 farms (58%) obtained biosecurity scores below the acceptability threshold. The average score was 57.57% (Table 4). Not surprisingly, most of paratuberculosis infected flocks showed insufficient biosecurity assessments results: 13 out of 19 (68%) infected farms had biosecurity values below 60% (Figure 3). 

The adoption of proper biosecurity measures is a key tool to prevent the introduction of MAP into the farm and to tackle its spread [5,7]. Therefore, also for the 15 biosecurity indicators a statistical analysis was performed, in order to explore possible association with paratuberculosis status. For two indicators a correlation was demonstrated: (i) “Contact with other animal species” and (ii) “Measures for preventing the entrance of strangers”.

Contact prevention with other animal species represents a general biosecurity measure on farms, which also applies to paratuberculosis, although the check-list indicator considers all species and is not focused on those that may be a source of MAP (e.g., ruminants). Similarly, preventing the entrance of strangers in the farm is a general biosecurity measure, therefore a correlation with this indicator and infected farms is not surprising.

Correlation with the other biosecurity indicators could not be demonstrated, probably due to the diffuse low level of biosecurity in all assessed flocks, a critical issue already reported in cattle farming [37].

Moreover, the assessment through checklists provides a “picture” at the moment of the visit to the farm. So, since paratuberculosis is a chronic disease, conditions that could have led to the entry and spread of the infection in the flocks could may not been highlighted at the moment of the assessment. For paratuberculosis control, the general biosecurity measures included by the checklist should be implemented with specific management procedures, such as feed milk replacers or pasteurized milk in infected farms, ensure at least the separation of test-positive and test-negative animals, organizing positive and negative groups, prevent manure contamination of feed and water. 

Our findings may be indicative of how some deficiencies in livestock management and biosecurity may favor the introduction and spread of communicable diseases, which, in turn, may affect some animal welfare parameters. In this exploratory study, the execution of a multiple model was dispensed with, since the study design, characterized by opportunistic sampling, is not suitable for this type of analysis.

Finally, in accordance with data reported in Italy in cattle [38], we observed that the mean year-long milk production is higher in MAP negative flocks (Table 3). Nevertheless, the high SD values suggest that this observation may not be significant. 

## 5. Conclusions

The results of this study confirm that paratuberculosis is present in northern Italy dairy goat farms. Because of possible biases related to low representativeness of the sample considered, this investigation was presented as exploratory.

Knowledge and surveillance by veterinarians and farmers are essential in order to counteract paratuberculosis impact on the economy of the farm, on the animal welfare and on public health. 

The animal welfare and biosecurity assessments through the described checklists proved to be an accurate and easy-to-use tool in the field, capable of identifying critical points and providing the farmers with indications to improve farm management. Our survey highlighted that, on the investigated dairy goat farms, the animal welfare level is on average acceptable, while more effort should be directed toward improving biosecurity levels. 

## Figures and Tables

**Table 1:** Indicators for welfare assessment (36), divided into “Area A: farm management and staff training”, “Area B: housing”, “Area C: animal-based measures” and based on a 2 or 3-point scale scoring system (1: insufficient status, 2: acceptable status, 3: excellent status).

**Area A—Management Factors**	**Scoring Scale**
1. Number of stockpersons	1, 2, 3
2. Experience and training of stockpersons	1, 2, 3
3. Animal grouping strategy	1, 2, 3
4. Inspection of the animals	1, 2, 3
5. Management of sick or injured animals	1, 2
6. Feeding strategy	1, 2, 3
7. Water provision	1, 2, 3
8. Cleanliness of water points	1, 2, 3
9. Cleanliness and hygiene of floor in walking areas and of bedding	1, 2, 3
10. Hygiene of milking procedures	1, 2, 3
11. Biosecurity measures	1, 2, 3
**Area B—Housing factors**	
12. Building and animal housing	1, 2
13. Presence of shelters in outdoor areas	1, 2, 3
14. Type of housing	1, 2, 3
15. Space availability in lying area (adult goats)	1, 2, 3
16. Space availability in lying area (young goats)	1, 2, 3
17. Space availability in lying area (bucks)	1, 2, 3
18. Space availability in lying area and feed bunk dimension (kids)	1, 2, 3
19. Feeding place dimension and accessibility	1, 2, 3
20. Functioning and number of water points	1, 2, 3
21. Facilities for sick animals	1, 2, 3
22. Milking machine or robot maintenance	1, 2, 3
23. Temperature and humidity	1, 2, 3
24. Lighting	1, 2
**Area C** **—** **Animal based measures**	
25. First-contact latency test	1, 2, 3
26. Isolated animals, staring into space and estranged from the environment	1, 2, 3
27. Body condition score	1, 2, 3
28. Cleanliness of the animals	1, 2, 3
29. Integument alterations	1, 2, 3
30. Lameness	1, 2, 3
31. Overgrown claws	1, 2, 3
32. Abscesses	1, 2, 3
33. Udder asymmetries	1, 2, 3
34. Annual mortality rate (adult goats)	1, 2, 3
35. Annual mortality rate (kids)	1, 2, 3
36. Mutilations	1, 2, 3

**Table 2:** Measures for the biosecurity assessment (15) based on a 2 or 3-point scale scoring system (1: insufficient status, 2: acceptable status, 3: excellent status).

Biosecurity Plan	Scoring Scale
1. Measures for rodent and insect fighting	1, 2, 3
2. Contact with other animal species	1, 2, 3
3. Measures for preventing the entrance of strangers	1, 2, 3
4. Measures for monitoring the entrance of regular visitors	1, 2, 3
5. Disinfection of vehicles entering the farm	1, 2, 3
6. Indirect–direct contacts between own animals and outside vehicles	1, 2
7. Collection disposal of fallen stock (dead livestock)	1, 2
8. Loading of live animals	1, 2
9. Purchase and/or movement of animals to outside the farm	1, 2, 3
10. Quarantine measures	1, 2, 3
11. Control and prevention of major infectious diseases	1, 2, 3
12. Health monitoring activities	1, 2
13. Control and prevention of mammary infections	1, 2, 3
14. Control and prevention of endo/ectoparasitosis	1, 2, 3
15. Monitoring and analysis of water sources	1, 2, 3

**Table 3:** Data about number of lactating goats and mean year-long milk production (expressed as kilograms per head per day) in seropositive (19) and seronegative (14) flocks.

	Mean	SD	Min	1st Quartile	Median	3rd Quartile	Max
Lactating goats	100	64	20	46	80	133	280
pos	111	75	20	47	83	138	280
neg	85	44	34	43	71	131	153
Milk production (Kg/head/day)	2.84	0.72	1.60	2.25	2.90	3.22	4.70
pos	2.69	0.7	1.6	2.1	2.9	3.26	4
neg	3.06	0.72	2	2.48	3	3.33	4.7

**Table 4:** Results obtained from welfare (Total, Area A, Area B and Area C) and biosecurity assessment in the 33 farms by paratuberculosis positive (19 farms) or negative (14 farms) serological status.

	Mean	Min.	1st Quartile	Median	3rd Quartile	Max.
**Total welfare**	68.64	39.56	60.16	66.14	78.88	90.70
pos	64.66	39.56	53.54	65.41	76.59	88.89
neg	74.03	58.50	63.16	77.21	83.63	90.70
**Area A ^1^**	67.92	41.29	56.14	67.20	78.72	96.16
pos	61.23	41.29	50.50	62.84	73.00	92.05
neg	76.99	54.73	66.20	78.72	88.34	96.16
**Area B ^2^**	60.36	37.21	51.24	57.08	68.43	93.59
pos	56.91	37.21	47.25	53.03	62.75	89.36
neg	65.03	46.99	51.78	63.19	78.11	93.59
**Area C ^3^**	70.41	30.00	56.50	69.21	82.93	100.00
pos	67.11	30.00	56.21	66.21	82.79	94.07
neg	74.89	52.7	64.75	76.11	84.36	100.00
**Biosecurity**	57.57	10.04	44.60	55.68	75.19	90.01
pos	53.08	10.04	40.86	51.99	74.79	89.76
neg	63.65	29.73	51.89	64.25	76.98	90.01

^1^ Farm management and staff training, ^2^ Housing factors, ^3^ Animal based measures.

**Table 5:** Statistical analysis of the indicators from the animal welfare and biosecurity assessment that showed a correlation with paratuberculosis seropositivity (*p*-value < 0.10).

Indicator	Score	Flocks (N)	Positive Flocks	Statistical Analysis
**Welfare**				
Animal grouping strategy	excellent	18	7	χ^2^ = 6.51 *p*-value = 0.039
	acceptable	13	11	
	insufficient	2	1	
Inspection of the animals	excellent	16	6	χ^2^ = 3.65 *p*-value = 0.056
	acceptable	17	13	
	insufficient	0	0	
Cleanliness and hygiene of floor in walking areas and of bedding	excellent	16	6	χ^2^ = 5.37 *p*-value = 0.068
	acceptable	16	12	
	insufficient	1	1	
Space availability in lying area (young goats)	excellent	11	5	χ^2^ = 4.64 *p*-value = 0.098
	acceptable	20	14	
	insufficient	2	0	
Annual mortality rate (adult goats)	excellent	9	4	χ^2^ = 6.18 *p*-value = 0.045
	acceptable	14	6	
	insufficient	10	9	
**Biosecurity**				
Contact with other animal species	excellent	12	4	χ^2^ = 11.64 *p*-value = 0.003
	acceptable	5	1	
	insufficient	16	14	
Measures for preventing the entrance of strangers	excellent	4	2	χ^2^ = 9.25 *p*-value = 0.01
	acceptable	20	8	
	insufficient	9	9	

## Data Availability

The data presented in this study are available on request from the corresponding author. The data are not publicly available due to privacy restrictions.

## Supplementary Materials

The following supporting information can be downloaded at: https://www.mdpi.com/article/10.3390/ani13142346/s1: Table S1: Details of breeds, management, number of lactating goats and mean year-long milk production. Table S2: Statistical analysis of the indicators from the animal welfare and biosecurity assessment associated with paratuberculosis seropositivity. Statistical analysis was not applicable when the same score was assigned to all the flocks.
