# Supplementary material for: Paratuberculosis, Animal Welfare and Biosecurity: A Survey in 33 Northern Italy Dairy Goat Farms
Source: Animals (Basel). 2023 Jul 18;13(14):2346. doi: 10.3390/ani13142346 (PMC10376716; doi:10.3390/ani13142346)
Supplement: Supplementary file 1 [file animals-13-02346-s001.zip › animals-2369429-supplementary.pdf]

Table S1. Details of breeds, management, number of lactating goats and mean year-long milk production.

Farm	Breed	Management	Lactating goats	Milk Production (Kg/day)
1	Chamos Coloured, Saanen,crossbreed	intensive	131	2.8
2	Chamois Coloured, Roccaverano	semi-intensive	115	3.16
3	Chamois Coloured, Roccaverano	semi-intensive	120	3.4
4	Chamois Coloured	intensive	65	4
5	Murciana	semi-intensive	30	2
6	Chamois Coloured	intensive	130	2.4
7	Saanen	intensive	44	3
8	Saanen, Nubiana, crossbreed	intensive	34	3.6
9	Saanen	intensive	83	3
10	Saanen	intensive	210	2.9
11	Chamois Coloured	intensive	38	2.94
12	Chamois Coloured	intensive	135	2.5
13	crossbreed	intensive	45	3
14	Saanen, crossbreed	intensive	130	1.6
15	Chamois Coloured, Roccaverano	intensive	250	3.26
16	Chamois Coloured, Saanen, Nera Verzasca	semi-intensive	182	2.1
17	Chamois Coloured, Roccaverano	semi-intensive	80	2.9
18	Chamois Coloured	intensive	66	2.7
19	Chamois Coloured	intensive	40	3
20	Chamois Coloured, Saanen, crossbreed	intensive	280	2.6
21	Chamois Coloured, Saanen	intensive	140	4.7
22	Saanen	intensive	120	2.2
23	Murciana	intensive	70	2.2
24	Chamois Coloured	semi-intensive	20	1.6
25	Chamois Coloured, Roccaverano	semi-intensive	110	3.17
26	Saanen	intensive	60	4
27	Chamois Coloured	intensive	65	1.7
28	Chamois Coloured, Saanen	intensive	153	3.8
29	Saanen	intensive	138	2.9
30	Chamois Coloured	intensive	47	2.3
31	Chamois Coloured	semi-intensive	34	2
32	Saanen	intensive	47	3.3
33	Saanen	intensive	75	3.1

Table S2. Statistical analysis of the indicators from the animal welfare and biosecurity assessment associated with paratuberculosis seropositivity. Statistical analysis was not applicable when the same score was assigned to all the flocks.

Indicators	Score	Flocks (N)	Positive Flocks	Statistical Analysis
**Welfare Area A**				
Number of stockpersons	excellent	17	9	$\chi^2 = 1.22$
	acceptable	11	6	*p*-value = 0.543
	insufficient	5	4	
Experience and training of stockpersons	excellent	15	8	$\chi^2 = 2.95$
	acceptable	14	10	*p*-value = 0.229
	insufficient	4	1	
Animal grouping strategy	excellent	18	7	$\chi^2 = 6.51$
	acceptable	13	11	*p*-value = 0.039
	insufficient	2	1	
Inspection of the animals	excellent	16	6	$\chi^2 = 3.65$
	acceptable	17	13	*p*-value = 0.056
	insufficient	0	0	
Management of sick or injured animals	acceptable	33	19	Not applicable
	insufficient	0	0	
Feeding strategy	excellent	22	10	$\chi^2 = 2.62$
	acceptable	11	9	*p*-value = 0.105
	insufficient	0	0	
Water provision	excellent	22	12	$\chi^2 = 0.02$
	acceptable	11	7	*p*-value = 0.901
	insufficient	0	0	
Cleanliness of water points	excellent	10	6	$\chi^2 = 0.71$
	acceptable	19	10	*p*-value = 0.701
	insufficient	4	3	
Cleanliness and hygiene of floor in walking areas and of bedding	excellent	16	6	$\chi^2 = 5.37$
	acceptable	16	12	*p*-value = 0.068
	insufficient	1	1	
Hygiene of milking procedures	excellent	17	8	$\chi^2 = 2.42$
	acceptable	10	6	*p*-value = 0.298
	insufficient	6	5	
Biosecurity measures	excellent	13	5	$\chi^2 = 3.6$
	acceptable	19	13	*p*-value = 0.166
	insufficient	1	1	
**Area B**				
Building and animal housing	acceptable	33	19	Not applicable
	insufficient	0	0	
Presence of shelters in outdoor areas	excellent	4	1	$\chi^2 = 0.75$
	acceptable	29	18	*p*-value = 0.386
	insufficient	0	0	
Type of housing	excellent	17	8	$\chi^2 = 0.82$
	acceptable	16	11	*p*-value = 0.364
	insufficient	0	0	
Space availability in lying area (adult goats)	excellent	7	2	$\chi^2 = 3.08$

	acceptable	15	10	p-value = 0.214
	insufficient	11	7	
Space availability in lying area (young goats)	excellent	11	5	χ^2 = 4.64
	acceptable	20	14	p-value = 0.098
	insufficient	2	0	
Space availability in lying area (bucks)	excellent	3	1	χ^2 = 0.88
	acceptable	28	17	p-value = 0.643
	insufficient	2	1	
Space availability in lying area and feed bunk dimension (kids)	excellent	7	3	χ^2 = 0.91
	acceptable	24	15	p-value = 0.636
	insufficient	2	1	
Feeding place dimension and accessibility	excellent	8	3	χ^2 = 2.12
	acceptable	21	14	p-value = 0.346
	insufficient	4	2	
Functioning and number of water points	excellent	10	6	χ^2 = 0.21
	acceptable	15	8	p-value = 0.899
	insufficient	8	5	
Facilities for sick animals	excellent	6	2	χ^2 = 3.76
	acceptable	14	7	p-value = 0.152
	insufficient	13	10	
Milking machine or robot maintenance	excellent	22	13	χ^2 = 0
	acceptable	11	6	p-value = 1
	insufficient	0	0	
Temperature and humidity	excellent	3	2	χ^2 = 0.15
	acceptable	28	16	p-value = 0.927
	insufficient	2	1	
Lighting	acceptable	33	19	Not applicable
	insufficient	0	0	
**Area C**				
First-contact latency test	excellent	28	15	χ^2 = 1.42
	acceptable	4	3	p-value = 0.492
	insufficient	1	1	
Isolated animals, staring into space and estranged from the environment	excellent	33	19	χ^2 = 0.76
	acceptable	0	0	p-value = 0.384
	insufficient	0	0	
Body condition score	excellent	26	13	χ^2 = 2.98
	acceptable	6	5	p-value = 0.226
	insufficient	1	1	
Cleanliness of the animals	excellent	27	15	χ^2 = 2.43
	acceptable	5	4	p-value = 0.296
	insufficient	1	0	
Integument alterations	excellent	19	9	χ^2 = 3.08
	acceptable	7	6	p-value = 0.214
	insufficient	7	4	
Lameness	excellent	30	17	χ^2 = 0.79
	acceptable	1	1	p-value = 0.672
	insufficient	2	1	
Overgrown claws	excellent	16	9	χ^2 = 0.5
	acceptable	9	6	p-value = 0.777

	insufficient	8	4	
Abscesses	excellent	12	7	$\chi^2 = 0.16$ *p*-value = 0.925
	acceptable	8	5	
	insufficient	13	7	
Udder asymmetries	excellent	19	11	$\chi^2 = 0$ *p*-value = 0.999
	acceptable	7	4	
	insufficient	7	4	
Annual mortality rate (adult goats)	excellent	9	4	$\chi^2 = 6.18$ *p*-value = 0.045
	acceptable	14	6	
	insufficient	10	9	
Annual mortality rate (kids)	excellent	15	6	$\chi^2 = 4.54$ *p*-value = 0.103
	acceptable	7	4	
	insufficient	11	9	
Mutilations	excellent	10	5	$\chi^2 = 0.04$ *p*-value = 0.844
	acceptable	23	14	
	insufficient	0	0	
**Biosecurity**				
Measures for rodent and insect fighting	excellent	5	2	$\chi^2 = 0.79$ *p*-value = 0.672
	acceptable	25	15	
	insufficient	3	2	
Contact with other animal species	excellent	12	4	$\chi^2 = 11.64$ *p*-value = 0.003
	acceptable	5	1	
	insufficient	16	14	
Measures for preventing the entrance of strangers	excellent	4	2	$\chi^2 = 9.25$ *p*-value = 0.01
	acceptable	20	8	
	insufficient	9	9	
Measures for monitoring the entrance of regular visitors	excellent	2	1	$\chi^2 = 0.85$ *p*-value = 0.655
	acceptable	26	16	
	insufficient	5	2	
Disinfection of vehicles entering the farm	excellent	1	1	$\chi^2 = 0.77$ *p*-value = 0.681
	acceptable	18	10	
	insufficient	14	8	
Indirect-direct contacts between own animals and outside vehicles	acceptable	16	8	$\chi^2 = 0.25$ *p*-value = 0.616
	insufficient	17	11	
Collection disposal of fallen stock (dead livestock)	acceptable	24	12	$\chi^2 = 1.09$ *p*-value = 0.297
	insufficient	9	7	
Loading of live animals	acceptable	21	13	$\chi^2 = 0.09$ *p*-value = 0.765
	insufficient	12	6	
Purchase and/or movement of animals to outside the farm	excellent	11	5	$\chi^2 = 1.02$ *p*-value = 0.599
	acceptable	16	10	
	insufficient	6	4	
Quarantine measures	excellent	12	6	$\chi^2 = 2.07$ *p*-value = 0.356
	acceptable	12	6	
	insufficient	9	7	
Control and prevention of major infectious diseases	excellent	14	7	$\chi^2 = 1.81$ *p*-value = 0.404
	acceptable	17	10	
	insufficient	2	2	
Health monitoring activities	acceptable	26	14	$\chi^2 = 0.16$ *p*-value = 0.686
	insufficient	7	5	

Control and prevention of mammary infections	excellent	14	8	$\chi^2 = 0.89$ *p*-value = 0.64
	acceptable	14	9	
	insufficient	5	2	
Control and prevention of endo/ectoparasitosis	excellent	24	13	$\chi^2 = 0.06$ *p*-value = 0.801
	acceptable	9	6	
	insufficient	0	0	
Monitoring and analysis of water sources	excellent	31	18	$\chi^2 = 0$ *p*-value = 1
	acceptable	0	0	
	insufficient	2	1	
